# Cost analysis of severe burn victims in Southwest China: A 7-year retrospective study

**DOI:** 10.3389/fpubh.2022.1052293

**Published:** 2023-01-09

**Authors:** Zhuo Zeng, Ning Li, Ling Yang, Xue Feng, Fangqing Zuo, Gaoxing Luo, Yizhi Peng, Zhiqiang Yuan

**Affiliations:** State Key Laboratory of Trauma, Burns and Combined Injury, Institute of Burn Research, Southwest Hospital, Third Military Medical University (Army Medical University), Chongqing, China

**Keywords:** health care economics, burns, hospitalization costs, epidemiology, medical consumables

## Abstract

**Background:**

Severe burn injury can be a life-threatening experience and can also lead to financial issues for suffers. The purpose of the current study was to analyze the direct hospitalization costs of severe burn inpatients in Southwest China.

**Methods:**

Data related to all inpatients admitted with severe burns [total body surface area (TBSA) ≥30%] pooled from 2015 to 2021 were reviewed retrospectively at the Institute of Burn Research of Army Medical University. Demographic parameters, medical economics, and clinical data were obtained from medical records.

**Results:**

A total of 668 cases were identified. The average age was 37.49 ± 21.00 years, and 72.3% were men. The average TBSA was 51.35 ± 19.49%. The median length of stay of inpatients in the burn intensive care unit was 14 [interquartile range (IQR): 5.0–34.8] days, and the median length of stay (LOS) was 41 (IQR: 22.0–73.8) days. The mortality rate was 1.6%. The median total cost was 212,755.45 CNY (IQR: 83,908.80–551,621.57 CNY) per patient varying from 3,521.30 to 4,822,357.19 CNY. The direct cost of scald burns was dramatically lower compared with that of other types of burns, with 11,213.43 to 2,819,019.14 CNY. Medical consumables presented the largest portion of total costs, with a median cost of 65,942.64 CNY (IQR: 18,771.86–171,197.97 CNY). The crucial risk factors for medical cost in our study were TBSA, surgical frequency, LOS, depth of burn, and outcome.

**Conclusion:**

We conclude that an effective burn prevention program, shorter hospital stays, and facilitating the healing of wounds should be focused on with tailored precautionary protocols to reduce the medical costs of inpatients with severe burns.

## Introduction

Severe burns are devastating injuries that are associated with considerable morbidity and mortality rates. However, as burn care levels have improved rapidly in recent years, an increasing number of patients with severe burns survive (1, 2). Advances in resuscitation, critical care, burn wound management, adequate supportive care, and rehabilitation have resulted in improved survival rates (3).

Unfortunately, the innovations in healthcare bring more costs, which may create enormous economic burdens for inpatients, their families, and society (4–6). Moreover, repeated operations, prolonged hospitalization, and long convalescence phases add to the high cost in the treatment of severe burns (7, 8). Therefore, the high costs arising from severe burns, a severe public health challenge, should warrant our special consideration. Collecting timely and accurate epidemiological data might be useful for developing prevention strategies for severe burns, improving treatment outcomes, and reducing the burden of medical expenses. However, there are few studies on the epidemiology and the medical costs in the treatment of severe burns in low- and middle-income countries. Most of the previous studies on severe burns pay more attention to vulnerable age groups or specific types of burns (9–11). Finding detailed information about the high costs of burn care can drive the search for cost-effectiveness. The purpose of our research was to analyze the epidemiology and associated medical costs associated with severe burn victims admitted to our burn center between 2015 and 2021 in Southwest China.

## Methods

### Data collection

This is a retrospective study only comprising patients with severe burns (TBSA ≥ 30%) admitted to the Institute of Burn Research of Southwest Hospital between 2015 and 2021. Our burn center is one of the most established and best burn centers in China, and is attached to the Third Military Medical University (Army Medical University) and serves most burn patients from Southwest China. Our burn center consists of 18 intensive care beds and 107 common ward beds, with three operating rooms, and one emergency care room (12). In addition, we also developed a pioneering and the largest burn database in China, offering convenient access to the data of burn patients (13). The following variables in our study were extracted and collected from medical records: demographic parameters, cause of burns, total burned surface area (TBSA), burn depth (% third degree and % second degree), patient outcome, length of hospital stay (LOS), inhalation injury, burn intensive care unit LOS, number of operations, and economic data. The medical costs we collected were grouped into 10 categories: medications, laboratory tests, physician examinations, treatment, surgery, anesthetic procedures, blood products, medical consumables, inpatient ward, and hospital care. The treatment fees included non-surgical wound management (such as dressing changes), treatment of complications, and nutritional support. Wound dressings were included in the medical consumables category. In addition, we collected the cost of rehabilitation therapies specifically, which were contained in some expense categories such as medical consumables and treatment, and were supported by rehabilitation physicians, including interventions, such as splinting, proper limb positioning, and laser therapy.

All patients in our burn center were treated according to a standard protocol that included first aid, fluid resuscitation, burn wound assessment and coverage, and supportive care such as infection control and nutrition therapy, diagnosis and treatment of injury complications, and rehabilitation. Patients were managed entirely in the Institute of Burn Research until they were discharged. The prognostic burn index (PBI) (14), the Abbreviated Burn Severity Index (ABSI) (15), and the Baux score (16) were analyzed for all severe burn patients.

Approval for our retrospective study was obtained from the Ethics Committee of Southwest Hospital of Army Medical University (No. KY2022202). Before analysis, the information and medical records of patients were anonymized and de-identified, and on account of the retrospective property of this study, the informed consent requirement was waived by us.

### Data analysis and statistics

The results of the continuous variables presented in tables and graphs are represented as mean ± standard deviation or median [interquartile range (IQR)]. The categorical data were expressed as counts and percentages. On the basis of normal/non-normal distribution, homogeneity test of variances, and types of the value we gathered, we chose the Student's *t*-test or the ANOVA to compare quantitative variables following normal distribution, and we used the Mann–Whitney *U*-test, the Kruskal–Wallis test, or the chi-square test for comparison of categorical variables or quantitative variables without normal distribution. Finally, the risk factors for medical costs of inpatients with severe burns were ascertained by multiple linear regressions. Data analysis was carried out in Microsoft Excel 2019 and SPSS Statistics 26.0. We regarded a *P* < 0.05 as statistically significant.

## Results

### Characteristics of the population

**Figure 1:**
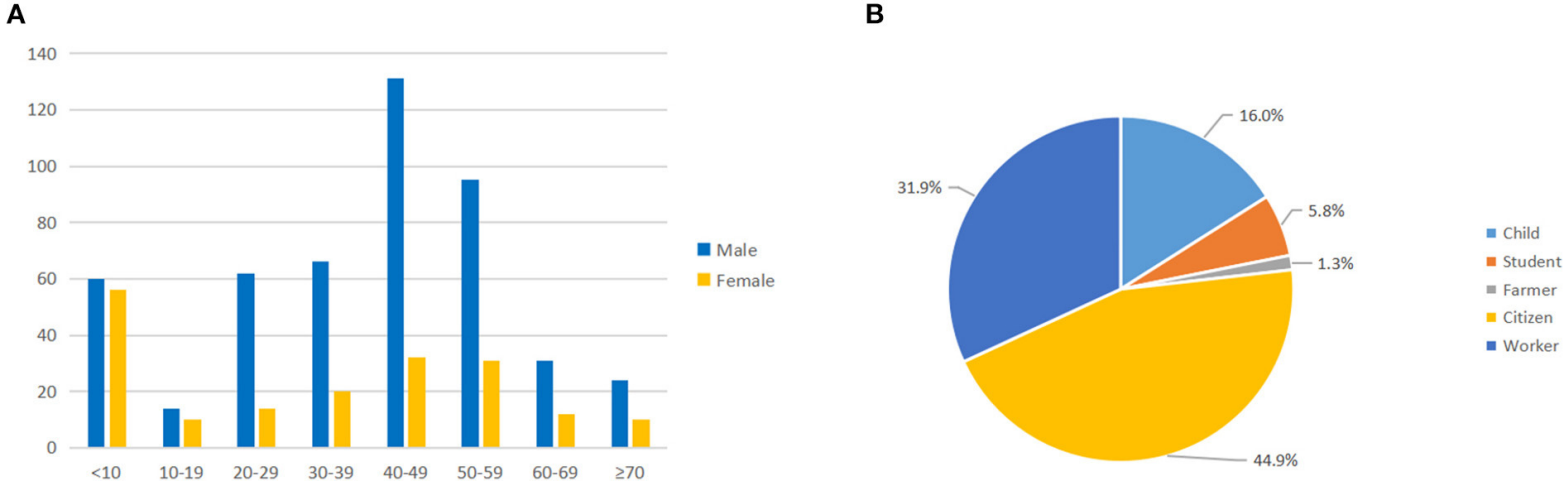
Age **(A)** and occupation **(B)** distribution of patients with severe burns.

The demographic characteristics of patients with severe burns are shown in Table 1. Over our research period, the number of patients (TBSA ≥ 30%) admitted to the Institute of Burn Research from 2015 to 2021 was 668. The inpatients with medical insurance accounted for 70.4% of the total severe burn inpatients during our study period. The average age of the 668 patients with severe burns was 37.43 ± 21.00 years (median 42), with 40 to 49 years old (24.4%, 163/668) being the most affected group, followed by 50–59 years old (18.9%, 126/668) (Figure 1A). Male patients accounted for 72.3% of our study. 59.0% of the severe burn patients lived in rural areas. The two most affected occupations were local residents (44.9%) and factory workers (31.9%) (Figure 1B). The median LOS was 41 days (IQR: 22.0–73.8 days). 90.9% of the patients required intensive care, and the median LOS in the burn intensive care unit was 14 days. In our study, 76.8% of patients underwent surgical operations, and the highest rate of surgical operations was obtained in 2021 (83.7%). The total response rate was 95.1%, the total cure rate was 72.5%, and the total mortality rate was 1.6%. The majority of inpatients in our study, 452 (67.7%), had full-thickness burn wounds.

**Table 1:** Characteristics of the study population between 2015 and 2021.

**Years**	**Cases (%)**	**Male *n* (%)**	**Age median (IQR)**	**Rural *n* (%)**	**Insurance *n* (%)**	**MV *n* (%)**	**Inhalation *n* (%)**	**Hemodialysis *n* (%)**	**Operation *n* (%)**	**LOS median (IQR)**	**ICU stay median (IQR)**	**Response rate *n* (%)**	**Cure rate *n* (%)**	**Mortality *n* (%)**
2015	112	73 (65.2%)	40.5 (20.5–50.8)	49 (43.8%)	79 (70.5%)	16 (14.3%)	29 (25.9%)	8 (7.1%)	69 (61.6%)	35.5 (16.0–56.0)	10.5 (5.0–20.8)	105 (93.8%)	81 (72.3%)	4 (3.6%)
2016	65	50 (76.9%)	39.0 (21.5–48.0)	56 (86.2%)	45 (69.2%)	11 (16.9%)	24 (36.9%)	4 (6.2%)	42 (64.6%)	36.0 (6.0–61.0)	12.0 (3.5–32.0)	61 (93.8%)	34 (52.3%)	1 (1.5%)
2017	106	75 (70.8%)	42.0 (20.3–54.0)	98 (92.5%)	70 (66%)	16 (15.1%)	24 (22.6%)	5 (4.7%)	84 (79.2%)	50.5 (22.0–87.3)	17.5 (6.8–35.3)	102 (96.2%)	73 (68.9%)	1 (0.9%)
2018	104	72 (69.2%)	45.0 (22.5–50.8)	57 (54.8%)	73 (70.2%)	22 (21.2%)	26 (25%)	26 (25%)	85 (81.7%)	43.5 (24.3–79.3)	13.0 (4.0–39.0)	95 (91.3%)	78 (75%)	2 (1.9%)
2019	92	74 (80.4%)	41.5 (24.3–51.0)	25 (27.2%)	68 (73.9%)	21 (22.8%)	34 (37%)	6 (6.5%)	72 (78.3%)	39.0 (21.3–71.5)	15.5 (5.0–40.5)	91 (98.9%)	66 (71.7%)	0 (0.0%)
2020	85	61 (71.8%)	41.0 (16.0–52.0)	45 (52.9%)	64 (75.3%)	22 (25.9%)	34 (40%)	34 (40%)	74 (87.1%)	53.0 (24.0–93.5)	23.0 (9.5–42.5)	82 (96.5%)	71 (83.5%)	2 (2.4%)
2021	104	78 (75%)	47.0 (31.0–55.0)	64 (61.5%)	71 (68.3%)	39 (37.5%)	58 (55.8%)	11 (10.6%)	87 (83.7%)	39.5 (22.3–84.8)	11.5 (3.0–29.5)	99 (95.2%)	81 (77.9%)	1 (1.0%)
Total	668	483 (72.3%)	42.0 (24.0–51.0)	394 (59%)	470 (70.4%)	147 (22%)	229 (34.3%)	94 (14.1%)	513 (76.8%)	41.0 (22.0–73.8)	14.0 (5.0–34.8)	635 (95.1%)	484 (72.5%)	11 (1.6%)
*P*-value		0.271^a^	0.182^b^	<0.001^a^	0.838^a^	0.001^a^	<0.001^a^	<0.001^a^	<0.001^a^	0.012^b^	0.002^b^	0.276^a^	0.002^a^	0.578^a^

IQR, interquartile range; MV, mechanical ventilation; LOS, length of stay; TBSA, total burned surface area.

a Chi-square test.

b Kruskal–Wallis test.

### Etiology

**Figure 2:**
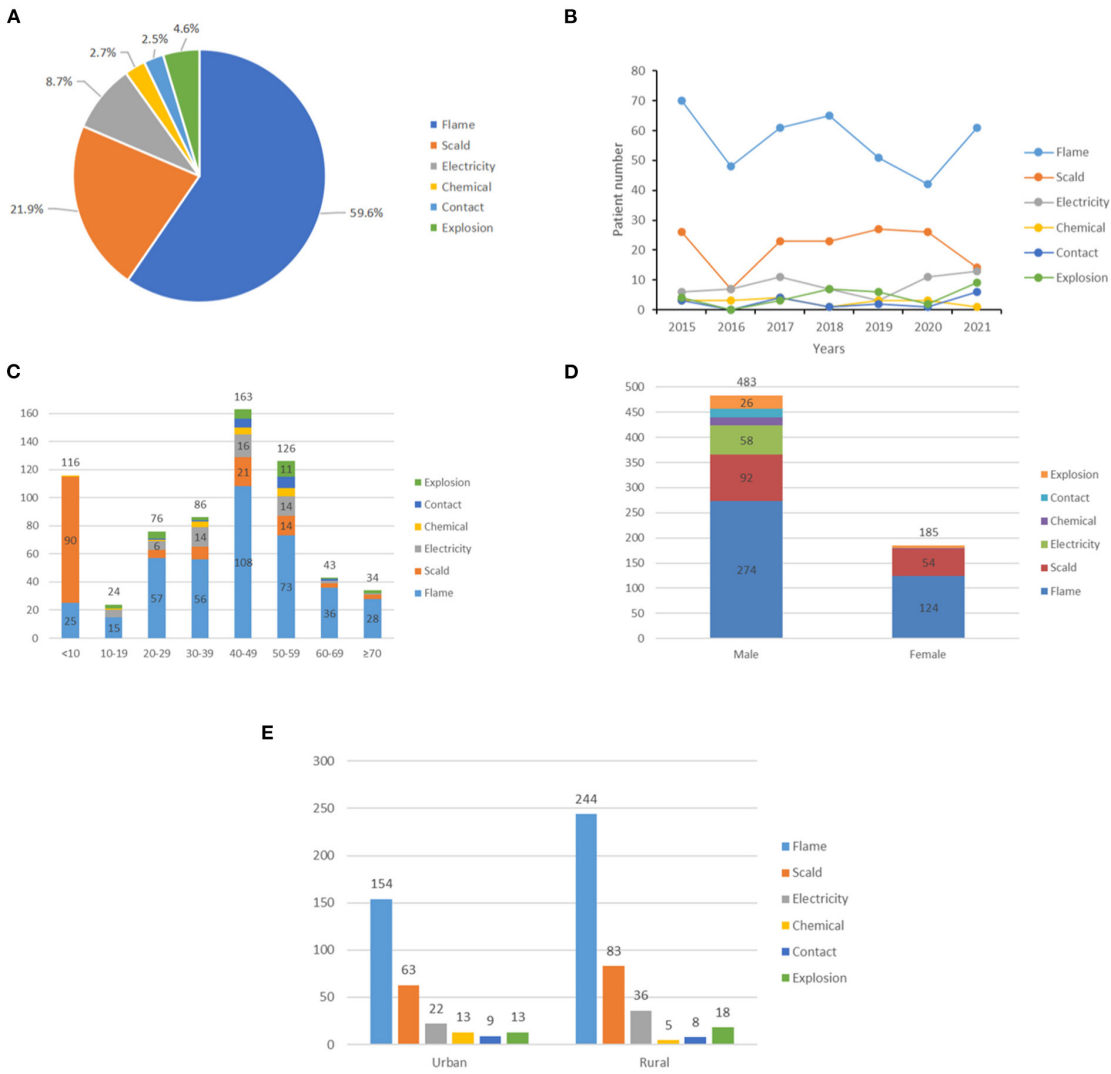
Etiological analysis. **(A)** Distribution of burn type. **(B)** Distribution of etiology by year. **(C)** Distribution of etiology by age. **(D)** Distribution of etiology by gender. **(E)** Distribution of etiology by area.

Regarding the etiology, the most frequent cause was flame, which was found in 398 (59.6%) cases. These were followed by scald, accounting for 21.9% of the inpatients. In addition, electricity, explosion, chemical, and contact burns accounted for 8.7, 4.6, 2.7, and 2.5%, respectively (Figure 2A). From 2015 to 2021, scald presented a decreasing trend, but a fluctuation was shown in the number of flame burns. In contrast, steady trends were shown in severe burns caused by the others (Figure 2B). In all age groups, the two major causes of severe burns were flame and scald, with scald mainly affecting children, and flame predominantly affecting juveniles, working-age populations, and older people (Figure 2C). Furthermore, the causes were different between male and female patients; electricity and contact burns were only observed in male patients, and scald burns had the lowest male-to-female ratio (Figure 2D). The proportion of patients with severe burns from rural areas (59.0%) was higher than that from urban areas (41.0%) (Figure 2E).

### Burn severity

The median TBSA was 45.0% (IQR: 35.0–63.8%). Inpatients with full-thickness burns accounted for the majority of our study (67.7%) based on maximum burn depth. The median ABSI was 9 (IQR: 8–12), ranging from 4 to 17. The median Baux score was 94 (IQR: 71–119), ranging from 31 to 170. The median PBI was 73 (IQR: 51–93), ranging from 2 to 148. Significant associations between burn severity scores and etiologies were found (Table 2; *P* < 0.001). It was dramatically lower for the Baux score, ABSI, and PBI of scald compared with other types of burn injuries. The Baux score, ABSI, and PBI of male patients were higher than that of female patients (Table 2; *p* < 0.001). In addition, the Baux score, ABSI, and PBI of the 0–20 year group were lower than those of the other age groups (Table 2; *p* < 0.001). Patients aged older than 60 years had the highest Baux score and PBI (Table 2; *p* < 0.001). However, there were no obvious differences detected in burn severity scores from 2015 to 2021.

**Table 2:** Burn severity of the study population.

	**TBSA median (IQR)**	**ABSI median (IQR)**	**Baux score median (IQR)**	**Prognostic burn Index median (IQR)**
**Etiology**				
Flame	49.5 (35.0–69.0)	10.0 (8.0–12.0)	101.0 (80.0–124.0)	79.0 (59.4–99.6)
Scald	39.0 (33.0–51.0)	7.0 (6.0–9.0)	55.0 (36.0–89.3)	29.8 (19.5–64.9)
Electricity	45.5 (35.0–60.0)	10.0 (8.8–11.0)	95.5 (78.8–110.5)	74.5 (63.9–88.5)
Chemical	46.5 (35.3–76.0)	9.0 (8.5–12.3)	97.0 (79.5–115.0)	77.3 (63.9–94.0)
Contact	46.0 (34.5–68.0)	10.0 (8.0–13.0)	96.0 (82.5–126.0)	77.5 (68.0–99.5)
Explosion	75.0 (45.0–91.0)	12.0 (9.0–15.0)	132.0 (90.0–150.0)	95.5 (64.5–118.00)
*P*-value	<0.001^a^	<0.001^a^	<0.001^a^	<0.001^a^
**Gender**				
Male	46.0 (35.0–68.0)	10.0 (8.0–12.0)	97.0 (76.0–123.0)	75.0 (55.5–94.50)
Female	43.0 (33.0–56.0)	8.0 (6.0–10.0)	85.0 (53.0–111.0)	67.5 (31.3–87.50)
*P*-value	<0.001^b^	<0.001^b^	<0.001^b^	<0.001^b^
**Age (years)**				
0–20	40.0 (32.0–51.3)	6.0 (5.0–8.0)	48.0 (36.0–60.5)	27.0 (19.4–42.5)
21–40	47.5 (35.0–68.0)	9.0 (7.0–12.0)	84.0 (71.0–111.0)	61.5 (51.5–79.0)
41–60	51.0 (37.0–72.0)	10.5 (9.0–13.0)	104.0 (89.0–129.8)	83.3 (71.5–102.5)
≥61	43.5 (34.8–51.3)	10.5 (10.0–12.0)	119.0 (110.0–133.8)	102.0 (90.9–111.6)
*P*-value	<0.001^a^	<0.001^a^	<0.001^a^	<0.001^a^
**Years**				
2015	45.0 (34.3–59.3)	9.0 (7.0–11.0)	93.5 (61.3–115.8)	70.5 (44.8–92.5)
2016	51.0 (35.5–72.5)	10.0 (8.0–12.0)	95.0 (76.0–123.5)	75.0 (56.3–97.5)
2017	45.0 (35.0–61.3)	9.0 (7.0–11.0)	94.0 (67.8–111.0)	72.0 (49.0–92.8)
2018	45.0 (35.0–62.8)	9.0 (7.0–12.0)	93.5 (62.0–116.5)	72.8 (48.3–89.0)
2019	44.0 (35.0–68.0)	9.0 (7.0–12.0)	89.5 (69.8–124.8)	68.5 (50.0–93.4)
2020	45.0 (33.5–59.5)	9.0 (7.5–12.0)	90.0 (67.0–127.0)	68.5 (43.8–96.0)
2021	46.0 (36.0–65.0)	10.0 (8.0–12.0)	102.0 (83.0–123.8)	79.3 (63.4–95.9)
*P*-value	0.510^a^	0.041^a^	0.069^a^	0.266^a^

IQR interquartile range.

a Mann–Whitney test.

b Kruskal-Wallis test.

### Analysis of medical cost

**Figure 3:**
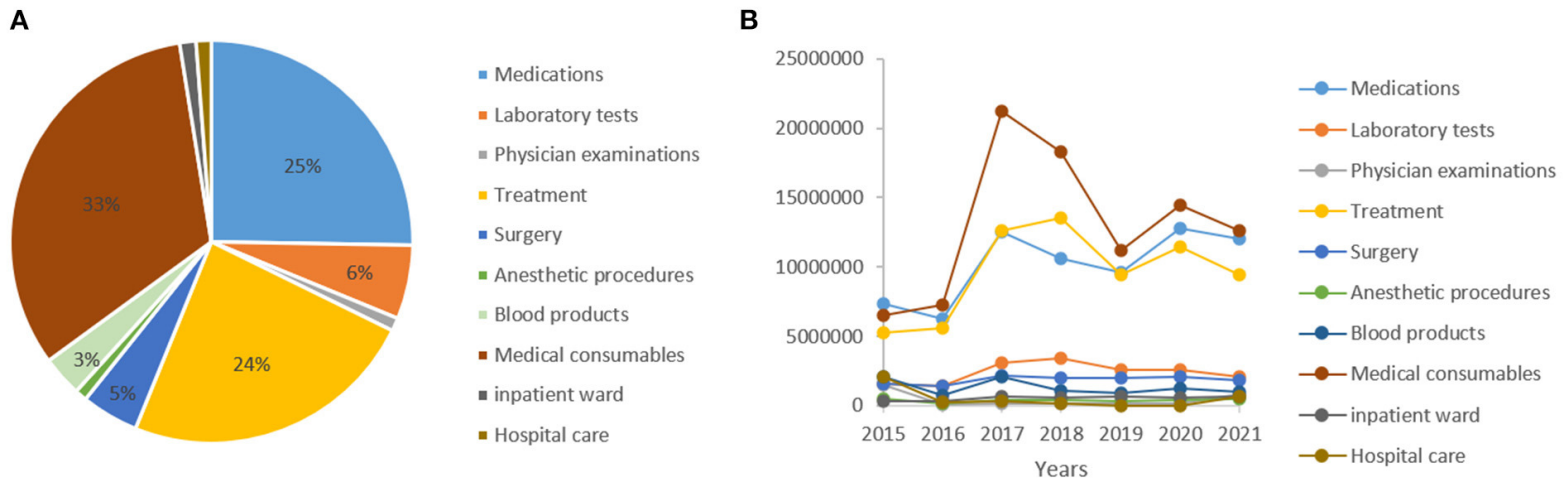
Cost analysis. **(A)** Distribution of expense category. **(B)** Distribution of expense category by year.

For the analysis of medical costs, the total cost for our study population at our burn center was 281,641,104.1 CNY, with a median total medical cost of 212,755.45 CNY (IQR: 83,908.80–551,621.57 CNY) per patient, varying from 3,521.30 to 4,822,357.19 CNY. The median daily cost was 5,862.15 CNY (IQR: 3,299.89–10,519.85 CNY), and the median cost per 1% of burn surface area was 4,773.65 CNY (IQR: 2,046.54–10,213.32 CNY). Of all cost categories, medical consumables accounted for the majority (33%), followed by medication fees (25%), therapeutic treatment fees (24%), laboratory tests (6%), surgery (5%), and blood products (3%) (Figure 3A). From 2015 to 2021, the total cost of medical consumables, medications, and treatment all showed an increasing trend. In contrast, steady trends were shown in other categories (Figure 3B).

**Figure 4:**
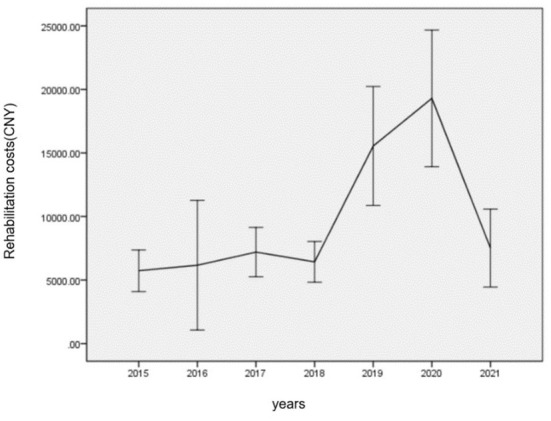
Distribution of rehabilitation costs stratified by year of the study population.

Overall, spending on medical consumables, medications, and therapeutic treatment was 91,542,173.94, 71,166,251.11, and 67,269,944.17 CNY, respectively, and the median daily cost of the three categories was 1,691.38 CNY (IQR: 792.68–3,322.29 CNY), 1,247.73 CNY (IQR: 608.68–2,477.64 CNY), and 1,288.44 CNY (IQR: 790.40–2,377.68 CNY), respectively (Table 3). Furthermore, 74.9% of the severe burn inpatients received rehabilitation therapies, and the total cost of rehabilitation was 6,326,200.20 CNY. Rehabilitation costs presented a roughly increasing trend from 2015 to 2021, which showed steady trends before 2018 and then sharply increased, reaching a peak in 2020 (Figure 4).

**Table 3:** Median daily cost and total cost distributions of patients classified by category.

**Category**	**Daily cost (CNY) median (IQR)**	**Total cost (CNY) median (IQR)**
Medications	1,247.73 (608.68–2,477.64)	47,585.87 (15,970.40–125,628.97)
Laboratory tests	325.75 (188.78–743.73)	12,247.35 (5,348.85–29,967.93)
Physician examinations	15.20 (4.38–51.43)	505.95 (144.80–2,353.48)
Treatment	1,288.44 (790.40–2,377.68)	48,461.58 (19,344.00–116,836.00)
Surgery	251.85 (30.53–472.60)	12,849.50 (369.60–29,673.78)
Anesthetic procedures	49.50 (12.94–90.16)	2,189.45 (52.80–5,406.28)
Blood products	123.96 (48.17–373.07)	5,012.00 (1,556.50–14,467.75)
Medical consumables	1,691.38 (792.68–3,322.29)	65,942.64 (18,771.87–171,197.97)
inpatient ward	90.00 (60.00–100.56)	3,190.00 (1,642.50–6,545.00)
Hospital care	23.36 (6.50–68.70)	870.50 (73.48–3,101.26)
Total	5,862.15 (3,299.89–10,519.85)	212,755.45 (83,908.80–551,621.57)

IQR, interquartile range; Values expressed as median (IQR).

Scald was the most inexpensive injury with a median total cost of 106,493.34 CNY (IQR: 48,331.44–214,249.58 CNY) and a median daily cost of 3,137.47 CNY (IQR: 2,214.31–4,612.88 CNY). On the other hand, explosion burns presented the most expensive, with a median total cost of 575,393.50 CNY (IQR: 246,321.16–1,506,502.97 CNY) and a median daily cost of 9,474.86 CNY (IQR: 6,201.64–28,775.47 CNY) (Table 4). The median total cost and median daily cost in patients with full-thickness burns were 338,022.55 CNY (IQR: 142,216.74–675,718.59 CNY) and 7,652.62 CNY (IQR: 4,650.47–13,940.73 CNY), respectively, which were found higher than those without full-thickness burns. With an increase in the burned area, an increasing trend in costs was shown. Patients with TBSAs of 71–80% presented the highest median total cost of 761,829.09 CNY (IQR: 400,954.66–1,196,200.97 CNY) and the highest median daily cost of 10,927.59 CNY (IQR: 7,932.50–16,556.83 CNY). However, in patients with a TBSA of >80%, a decreasing trend in medical costs was observed. When analyzing the costs in relation to gender, the median total cost and median daily cost for female inpatients were found lower than in male inpatients (*p* < 0.001; Table 4). When the costs were stratified by age, the median total cost, and median daily cost in patients aged 21–40 and 41–60 years were significantly higher compared with patients in other age categories (Table 4; *p* < 0.001). Moreover, the LOS showed significant differences among different causes of burn, burn depth, ages, TBSAs, and the number of surgical operations. As shown in Table 5, the median total costs of inpatients who received surgery are significantly higher than those of inpatients without surgery (*p* < 0.001). In addition, the median total cost and median daily cost of inpatients with TBSA of ≤50 were found the lowest in our study (*p* < 0.001). No significant difference in LOS was found between female and male patients (Table 4).

**Table 4:** Analysis of LOS and cost, classified by TBSA, etiology, age, gender, full-thickness, burns surgery numbers and year.

	**LOS (days) median (IQR)**	**LOS/TBSA (days) median (IQR)**	**Cost (CNY) median (IQR)**	**Cost/TBSA (CNY) median (IQR)**	**Daily cost (CNY) median (IQR)**
**Etiology**					
Flame	39.5 (17.0–69.3)	0.9 (0.4–1.5)	241,126.26 (88,338.16–529,448.10)	5,036.21 (2,042.38–9,762.27)	6,889.16 (4,040.22–12,444.26)
Scald	34.5 (22.0–54.0)	0.9 (0.6–1.2)	106,493.34 (48,331.44–214,249.58)	2,819.92 (1,402.03–4,620.39)	3,137.47 (2,214.31–4,612.88)
Electricity	70.0 (39.0–103.5)	1.4 (1.0–2.1)	501,006.66 (173,421.93–904,347.67)	9,543.80 (5,024.00–15,573.51)	6,603.60 (4,647.43–10,498.78)
Chemical	77.0 (36.5–114.5)	1.4 (0.8–1.9)	456,155.98 (127,989.86–979,647.83)	9,065.52 (3,346.49–15,395.05)	6,039.43 (3,378.49–8,874.84)
Contact	92.0 (44.5–134.0)	1.8 (1.1–3.1)	411,919.00 (121,888.58–811,787.87)	9,897.01 (3,340.52–21,968.06)	5,208.95 (3,174.86–9,004.55)
Explosion	56.0 (28.0–100.0)	1.2 (0.5–2.0)	575,393.50 (246,321.16–1,506,502.97)	8,654.00 (5,504.76–20,046.86)	9,474.86 (6,201.64–28,775.47)
*P*-value	<0.001^a^	<0.001^a^	<0.001^a^	<0.001^a^	<0.001^a^
**Gender**					
Male	42.0 (23.0–79.0)	1.0 (0.5–1.6)	252,365.68 (101,951.31–626,099.21)	5,261.34 (2,498.37–11,372.62)	6,454.03 (3,563.71–11,339.45)
Female	38.0 (18.0–62.0)	1.0 (0.4–1.4)	145,515.45 (53,898.22–347,728.52)	3,538.59 (1,376.27–8,093.54)	4,588.74 (2,558.36–8,827.09)
*P*-value	0.028^b^	0.381^b^	<0.001^b^	<0.001^b^	<0.001^b^
**Age (years)**					
0–20	37.5 (22.0–61.3)	1.0 (0.6–1.4)	110,428.65 (55,025.80–272,307.91)	2,946.25 (1,521.78–5,676.80)	3,375.96 (2,362.40–5,361.35)
21–40	42.5 (22.0–80.0)	1.0 (0.6–1.5)	247,222.65 (105,595.65–595,560.27)	5,022.54 (2,548.91–11,341.62)	6,129.76 (3,370.61–10,651.84)
41–60	47.5 (24.0–93.0)	1.1 (0.5–1.7)	315,188.39 (123,060.26–676,696.60)	6,348.73 (3,215.72–12,747.70)	6,851.83 (4,188.50–12,879.10)
≥61	24.0 (5.0–52.3)	0.6 (0.1–1.2)	139,952.03 (50,841.97–252,693.38)	3,094.99 (1,237.13–6,574.52)	7,904.97 (4,305.40–14,026.31)
*P*-value	<0.001^a^	<0.001^a^	<0.001^a^	<0.001^a^	<0.001^a^
**TBSA**					
≤40	35.0 (21.3–50.8)	1.0 (0.6–1.4)	105,691.15 (53,047.09–193,823.80)	3,259.67 (1,540.66–5,833.13)	3,346.22 (2,319.38–4,855.49)
41–50	45.0 (24.0–71.0)	1.0 (0.5–1.6)	223,135.20 (96,299.49–441,131.05)	4,958.56 (2,239.52–9,711.69)	5,735.66 (3,521.30–8,713.51)
51–60	58.5 (35.0–97.8)	1.1 (0.6–1.9)	406,848.99 (184,777.46–655,607.55)	7,617.55 (3,443.73–12,132.07)	7,559.20 (4,946.32–10,250.26)
61–70	71.5 (35.5–105.8)	1.1 (0.6–1.6)	484,573.30 (297,535.23–908,750.46)	7,024.48 (4,365.16–13,520.43)	9,472.80 (5,099.91–14,222.34)
71–80	71.0 (34.3–118.0)	0.9 (0.4–1.6)	761,829.09 (400,954.66–1,196,200.97)	10,392.17 (5,099.16–15,952.45)	10,927.59 (7,932.50–16,556.83)
81–90	31.0 (10.5–131.5)	0.4 (0.1–1.5)	611,922.45 (244,262.79–1,721,986.19)	7,199.09 (2,843.75–19,456.79)	15,036.36 (10,256.86–20,539.80)
91–100	18.5 (3.3–85.0)	0.2 (0.0–0.9)	404,743.21 (53,792.78–1,663,311.74)	4,350.72 (566.24–17,634.51)	20,906.69 (11,919.01–31,420.63)
*P*-value	<0.001^a^	<0.001^a^	<0.001^a^	<0.001^a^	<0.001^a^
**Full-thickness burns**					
With	51.0 (22.0–90.0)	1.1 (0.4–1.7)	338,022.55 (142,216.74–675,718.59)	6,514.62 (3,188.53–12,306.21)	7,652.62 (4,650.47–13,940.73)
Without	33.5 (21.0–49.8)	0.8 (0.6–1.2)	98,598.33 (49,623.01–192,591.61)	2,713.95 (1,411.26–4,585.25)	3,164.32 (2,239.69–4,863.68)
*P*-value	<0.001^b^	0.028^b^	<0.001^b^	<0.001^b^	<0.001^b^
**Surgery no**.					
0	21.0 (7.0–39.0)	0.6 (0.2–1.0)	72,090.91 (39,592.49–208,750.99)	1,840.37 (975.75–4,525.83)	5,082.75 (2,511.51–10,831.79)
1	32.0 (23.5–42.0)	0.9 (0.6–1.2)	130,998.86 (88,588.95–223,096.71)	3,182.72 (2,185.73–4,377.94)	4,091.28 (2,697.61–6,948.34)
2	49.0 (36.0–59.8)	1.2 (0.8–1.5)	249,768.78 (152,993.52–418,159.23)	5,088.43 (3,862.39–8,166.78)	5,173.68 (3,593.48–7,882.08)
3	59.0 (44.0–85.5)	1.3 (1.0–1.7)	385,723.35 (257,948.34–581,130.51)	7,712.48 (5,917.54–11,211.36)	5,714.73 (4,216.06–8,128.87)
≥4	97.0 (68.5–130.0)	1.6 (1.2–2.2)	896,574.59 (553,163.25–1,354,737.49)	14,385.67 (9,441.75–20,822.29)	9,158.93 (6,012.91–13,509.14)
*P*-value	<0.001^a^	<0.001^a^	<0.001^a^	<0.001^a^	<0.001^a^
**Years**					
2015	35.5 (16.0–56.0)	0.9 (0.4–1.2)	156,437.10 (64,342.37–310,213.18)	3,917.53 (1,529.14–6,412.11)	5,145.85 (2,794.44–8,621.89)
2016	36.0 (6.0–61.0)	0.9 (0.1–1.3)	165,396.03 (59,996.21–428,190.68)	3,404.21 (1,410.81–8,122.18)	5,749.74 (3,619.66–15,212.93)
2017	50.5 (22.0–87.3)	1.1 (0.5–1.9)	285,855.03 (127,846.38–679,742.34)	6,085.36 (3,037.16–12,244.03)	6,561.85 (3,815.69–11,841.61)
2018	43.5 (24.3–79.3)	1.0 (0.6–1.7)	214,409.75 (97,349.82–781,383.35)	4,941.48 (2,202.56–12,840.33)	5,951.74 (3,513.14–12,360.20)
2019	39.0 (21.3–71.5)	0.9 (0.6–1.4)	183,509.29 (54,487.58–532,631.92)	4,504.32 (1,554.29–9,446.56)	5,055.42 (2,943.55–9,740.33)
2020	53.0 (24.0–93.5)	1.2 (0.6–1.6)	285,014.34 (133,044.27–655,983.16)	6,909.20 (2,926.83–12,107.40)	5,896.89 (3,348.35–10,430.20)
2021	39.5 (22.3–84.8)	1.0 (0.5–1.5)	208,901.42 (94,397.38–612,183.60)	4,238.83 (2,465.20–10,854.09)	5,752.08 (3,187.09–10,743.44)
*P*-value	0.012^a^	0.012^a^	0.001^a^	<0.001^a^	0.11^a^

IQR, interquartile range; LOS, length of stay; TBSA, total burned surface area.

a Mann–Whitney test.

b Kruskal–Wallis test.

**Table 5:** Analysis of cost, classified by with or without surgery and burn area.

	**Cost (CNY) median (IQR)**	**Cost/TBSA (CNY) median (IQR)**	**Daily cost (CNY) median (IQR)**
**Surgery**			
Surgical group	349,624.07 (165,396.03–666,589.90)	6,895.48 (4,058.27–12,691.13)	6,238.16 (39,27.64–10,446.93)
Non-surgical group	72,090.91 (39,592.49–208,750.99)	1,840.37 (975.75–4,525.83)	5,082.75 (2,511.51–10,831.79)
*P*-value	<0.001^b^	<0.001^b^	0.003^b^
**TBSA**			
30–50	127,077.42 (57,786.17–253,459.37)	3,568.72 (1,631.75–6,895.48)	3,887.07 (2,502.52–6,144.01)
50–80	487,026.36 (234,212.72–903,778.09)	8,196.20 (4,095.06–13,509.33)	8,568.56 (5,408.06–13,225.48)
80–100	551,961.59 (156,123.37–1,644,686.26)	6,203.71 (1,714.74–18,092.51)	15,975.76 (11,311.89–25,559.81)
*P*-value	<0.001^a^	<0.001^a^	<0.001^a^

a Mann–Whitney test.

b Kruskal–Wallis test.

Table 6 shows the risk factors correlated with total medical cost by multiple linear regressions. The risk factors for the regression model contained the number of surgeries, intensive care unit LOS, length of stay (LOS), TBSA, inhalation injury, depth of burn, outcome, gender, age, and etiologies. We used natural logarithm (ln) transformation to evaluate the total medical cost. Among these factors, we noticed that the highest total cost could be yielded by the longer LOS (standardized regressio coefficient = 0.391; *p* < 0.001), followed by the higher frequency of surgical operations (standardized coefficient = 0.294, *P* < 0.001), larger TBSA (standardized regression coefficient = 0.254; *p* < 0.001), better outcomes (standardized regression coefficient = 0.148, *P* < 0.001), and third-degree burns (standardized regression coefficient = 0.072, *P* = 0.024).

**Table 6:** Risk factors of total medical cost, analyzed by multiple linear regression.

	**Unstandardized β coefficients**	**Standardized β coefficients**	***t***	***P*-value**
More surgeries	0.136	0.294	9.526	<0.001
Larger TBSA	0.016	0.254	8.618	<0.001
Longer LOS	0.008	0.391	12.861	<0.001
Better outcomes	0.861	0.148	5.518	<0.001
Full-thickness burns	0.203	0.072	2.261	0.024
**Etiology**				
Flame	−0.209	−0.079	−2.386	0.017
Scald	−0.400	−0.126	−3.475	0.001

## Discussion

This study mainly focused on the clinical characteristics and direct costs of inpatients with severe burns from 2015 to 2021 in Southwest China to further examine the economic features of severe burns and assist with the optimization of economic burden in severe burn management. The population incorporated in our research showed general features consistent with other epidemiologic studies on this issue (17, 18). Most of the patients with severe burns included in our study were male. The discrepancy in gender may be associated with the different divisions of labor between men and women in family and society. In China, men are more likely to work in dangerous fields with a high risk of burn injuries. Consistent with other reports (19), our research revealed that most of the inpatients with severe burns were aged 40–49 years old, suggesting that working-age populations were more likely to suffer severe burns. In our study, people who lived in rural areas were more likely to sustain severe burns, which coincided with other studies (20). Thus, there exist differences between rural and urban residents in risk factors of lifestyle related to burning. In addition, local residents accounted for the majority of inpatients with severe burns, and workers were the second most frequent victims in the research. There are two reasons for this. First, some residents had poor safety awareness. Second, less attention to production safety was paid by some enterprises, and some of them lacked safe facilities in order to reduce production costs. Therefore, public awareness should be enhanced, and rules for standardizing working safety should be implemented under the supervision of the government. Similar to the findings of previous reports (21, 22), the primary cause of severe burns was flame, which chiefly arises from fireworks, alcohol, hot oil, natural gas, and firewood. However, scald was the primary cause of severe burns for children, which mainly arise from hot water, hot foods, and hot steam. Thus, pertinent precautionary measures ought to be considered depending on the patient's age.

The present study showed that 67.7% of the severe burn inpatients had full-thickness burns, and significant differences were detected in burn severity scores in etiologies, genders, and ages. However, the mortality rate was 1.6%, which was lower than those of previous reports at other burn centers (22–24). The findings of our study could be interpreted as follows. First, based on the limitation of economic conditions and lack of medical insurance, many patients with low income chose to discharge themselves against the advice of physicians and did not die at the hospital. Second, improvement was achieved in the treatment level for severe burns at our center.

The median LOS in our study was 41 days (IQR: 22.0–73.8 days), which was longer than those in other reports (10, 13). There are several possible explanations for the difference. First, due to advanced medical conditions, hospitalized patients with severe burns who were transferred from other medical institutions were often received in our burn center. Second, inpatients with severe burns preferred receiving rehabilitation therapies and plastic surgery at our burn center after their burn wounds had healed. Our results also showed that the LOS was correlated with etiology, gender, and age, comparable with other reports (9, 25). Further analysis showed that the LOS and LOS/TBSA in the age group older than 61 years were shorter than other age groups, differing from other studies (25, 26). This might be because older people did not choose to stay at the hospital for rehabilitation therapies and their requirements for quality of life has been ignored.

In our study, the median cost of severe burns was 212,755.45 CNY (IQR: 83,908.80–551,621.57 CNY) per patient, 5,862.15 CNY (IQR: 3,299.89–10,519.85 CNY) per day, and 4,773.65 CNY (IQR: 2,046.54–10,213.32 CNY) per 1% of burn surface area, which was higher compared with those obtained in studies performed in other developing countries. For instance, a study from India showed that the cost was US $1,060.52 per patient and US $134.96 per day (27). In a study conducted in Brazil, the cost of victims with severe burns was US $39,594.90 per patient and US $1,330.48 per day (28). A burn cost study from Turkey suggested the mean total cost was US $15,250 per patient (29). In Malawi, a report showed a total cost of US$ 559.85 per patient and US $387.42 per day (30). In Iran, the hospital cost for all patients was about US $2,766 (31). However, a systematic review suggested that the median medical cost was US $44,024 per burn victim in high-income countries (32), which was higher than that obtained in our study. In China, there have been some published studies focusing on the medical cost of burns. In central China, a study reported that the median cost of pediatric burn inpatients was US $1,511 (33). Another study conducted in Wuhan suggested that the mean cost of pediatric burns was 11,210.76 and 1,630 CNY per % TBSA (34), which was lower than those in our study. In a study carried out at a burn center in Shanghai, the mean cost of burns in elderly patients was US $3,346.88, and the hospitalization cost of patients with extensive burns was US $5,176.21 (35), which was lower than the cost in our study, since the rehabilitation cost was not included. In South Central China, the median cost of pediatric burn patients in rural areas was US $2,139.48, compared with US $1,547.20 for pediatric burn patients in urban areas (36). Previous studies in China paid more attention to vulnerable populations or specific types of burns but were different from our study, and none of the other studies were related to the cost of severe burns. The difference in cost between our burn center with others might be related to the year of the report, the economic level of the country, burn severity, treatment method, and criteria of inclusion and exclusion.

Our report stated that medical consumables made up the majority of the direct medical charges, and an increasing trend was shown in medical consumables. This might be explained that inpatients with severe burns need more wound dressings to cover wounds than inpatients with minor burns during dressing changes, and most of the dressings used in clinical are imports so they tend to be expensive. However, medications or blood products were reported as the largest proportion of expenses in other studies (3, 28). In our burn center, the cost of blood products was listed as follows: red blood cells suspension (pRBC): 210 CNY/unit or 200 ml, fresh frozen plasma (FFP): 40 CNY/100 ml, and apheresis platelet (PLT): 1,600 CNY/unit. The price of different dressings included in the medical consumables ranges from 80 to 580 CNY/piece. The use of blood products is mainly in the early post-burn and perioperative periods. When a patient's condition has stabilized, blood products are usually unnecessary, but dressings are still needed until their wounds have healed. In addition, the quantity of wound dressings used in the dressing change process is based on the wound size. Therefore, the cost of blood products is significantly lower than medical consumables in this study. How to avoid unreasonable use of medical consumables and optimize the cost structure of hospitalization is still a challenge for us.

With the development of society and the improvement of the medical insurance system, people have gradually realized that burn rehabilitation is essential to help victims with severe burns enhance their quality of life and return to earlier to work. Our results showed that 74.9% of the inpatients with severe burns received rehabilitation therapies, and the cost of rehabilitation presented a roughly increasing trend from 2015 to 2021, mostly because our burn center had established a dedicated team for rehabilitation since 2011 (37), and the team consisted of doctors specialized in rehabilitation, nurses, rehabilitation therapists, and a psychological counselor. Thus, increasingly, early rehabilitation treatment was adopted by more inpatients with severe burns. However, due to the impact of COVID-19 (38–40), inpatients with burns were advised to receive outpatient rehabilitation after they were discharged from our burn center, so our results showed that the cost of rehabilitation was lower in 2021 than in 2020.

Different from other reports (28), scald was the most inexpensive injury in our study, with a median total cost of 106,493.34 CNY (IQR: 48,331.44–214,249.58 CNY) and median daily cost of 3,137.47 CNY (IQR: 2,214.31–4,612.88 CNY). However, the cost of scald burns was still higher in our studies than in others (28). The mean cost associated with scald in Turkey was (US $8,894.00 ± 5,694.00) (29). The costs of inpatients with third-degree burns were found clearly higher than inpatients without third-degree burns. Conservative protocols were not suitable for inpatients with third-degree burns, which suggested that inpatients with third-degree burns needed more surgeries and longer LOS in contrast to inpatients without third-degree burns, and the total costs finally increased.

When analyzing the relationship between costs and TBSA, we found that higher costs of severe burns were associated with a larger TBSA, comparable with other reports (31). However, a decreasing trend in medical costs for patients with TBSA of >80% was observed in our study, which was also mentioned in a study in the Netherlands (41) and probably could be explained by the following reasons. On the one hand, of the patients presenting with TBSA of >80%, there might have been higher levels of mortality. On the other hand, there were patients who could not pay for subsequent treatment and discharged themselves against the advice of physicians. In the present study, it was found that the costs for patients aged 21–40 and 41–60 years were clearly higher than the costs in other age groups. One possibility was that through commercial insurance, working-age populations suffering from expensive medical costs was avoided. Another possibility was that working-age patients were focused on making money to support their families, so they were more eager to receive rehabilitation and plastic surgery, and, as a result, their medical costs increased. It was shown in our study that longer LOS played the biggest role in total medical expenses, followed by an increased surgical frequency, larger TBSA, better outcomes, and third-degree burns. Therefore, to reduce the medical costs of severe burns, attention should be paid to effective burn prevention programs, timely wound healing, and reducing LOS in future intervention protocols (41).

Based on the aforementioned results, it can be said that the costs of patients with severe burns were indeed expensive. Fortunately, the system of medical insurance has been improving gradually in China, thus the portion of medical costs reimbursed by commercial or public medical insurance for victims of severe burns has increased (42–44). In addition, a charity foundation known as Chunmiao Charities Aid Foundation for Burned Children was established in our center in 2012 to support child burn victims from poor families (12). Moreover, poor patients with severe burns may be able to raise money for medical costs through networking platforms. In this way, the medical cost and family burden of patients with severe burns can be reduced. Although the government in China has established several insurance programs that have enabled China to achieve near nationwide coverage of medical insurance, many patients with severe burns came to our burn center for medical care without medical insurance, and expensive out-of-pocket spending became a serious issue to face. This brought to our attention a medical insurance system that needs to be improved by the government in China to continue to help patients with severe burns.

It should be mentioned that our study has the following limitations. First, our study used direct charges instead of real costs for cost analysis. In fact, indirect costs were included in the real costs, which might have had an impact on cost calculations and conclusions, because these could account for about 20% of the real costs (41). However, it was difficult to determine indirect costs, and few of them were mentioned in the studies. Second, only inpatients with severe burns were included in our study; the costs of inpatients with TBSA <30% were excluded. Hence, this study is only representative of patients with severe burns. Third, our study only reflected the costs of patients with severe burns in Southwest China, since patients included in our study were admitted to a single burn center. However, special attention should be given to the fact that our study was the only Chinese research related to the direct medical expenses of inpatients with severe burns at a professional burn institution. Therefore, our study of medical costs in Southwest China could serve as a helpful example for the rest of our country and other countries, and the findings could provide valuable information to improve the cost-effectiveness of burn care and healthcare policy.

## Conclusion

Our study analyzed the clinical characteristics and medical costs of severe burns in Southwest China from 2015 to 2021. Our results showed that the medical expenses of severely burned inpatients were high at our burn center. Medical consumables presented the largest fraction of the total costs. Furthermore, the considerable risk factors for cost were the TBSA, surgical frequency, LOS, depth of burn, and outcome, and protocols of individualized intervention should be created on the strength of interrelated risk factors, such as timely wound healing and shortening the LOS.

## Data availability statement

The original contributions presented in the study are included in the article/supplementary material, further inquiries can be directed to the corresponding author.

## Ethics statement

The studies involving human participants were reviewed and approved by the Ethics Committee of Southwest Hospital of Army Medical University. Written informed consent for participation was not required for this study in accordance with the national legislation and the institutional requirements.

## Author contributions

ZZ, NL, and ZY conceived of and designed the study. LY, XF, FZ, GL, and YP prepared the study and acquired data. ZZ and NL participated in statistical analysis. ZZ, NL, and ZY drafted the manuscript. All authors had read and approved the manuscript.
